# Heat treatment of quinoa (*Chenopodium quinoa Willd*.) albumin: Effect on structural, functional, and *in vitro* digestion properties

**DOI:** 10.3389/fnut.2022.1010617

**Published:** 2022-09-15

**Authors:** Chao Yang, Xijin Zhu, Zhaoyun Zhang, Farong Yang, Yuming Wei, Zhen Zhang, Fumin Yang

**Affiliations:** ^1^College of Food Science and Engineering, Gansu Agricultural University, Lanzhou, China; ^2^Animal Husbandry, Pasture and Green Agriculture Institute, Gansu Academy of Agricultural Sciences, Lanzhou, China

**Keywords:** quinoa, albumin, heat treatment, structural, functional properties

## Abstract

Quinoa seeds are rich in protein, polyphenols, phytosterols, and flavonoid substances, and excellent amino acid balance that has been revisited recently as a new food material showing potential applied in fitness and disease prevention. Heat treatment is one of the most effective strategies for improving the physiochemical characteristics of a protein. However, research examining the effects of temperature on quinoa albumin (QA) properties is limited. In this study, QA was subjected to thermal treatment (50, 60, 70, 80, 90, 100, and 121°C). SDS−PAGE revealed that QA is composed of several polypeptides in the 10−40 kDa range. Amino acid (AA) analysis showed that the branched-chain amino acids (BCAAs), negatively charged amino acid residues (NCAAs), and positively charged amino acids (PCAAs) contents of QA were more than double that of the FAO/WHO reference standard. Additionally, heating induced structural changes, including sulfhydryl-disulfide interchange and the exposure of hydrophobic groups. Scanning electron microscopy demonstrated that the albumin underwent denaturation, dissociation, and aggregation during heating. Moreover, moderate heat treatment (60, 70, and 80°C) remarkably improved the functional properties of QA, enhancing its solubility, water (oil) holding capacity, and emulsification and foaming characteristics. However, heating also reduced the *in vitro* digestibility of QA. Together, these results indicate that heat treatment can improve the structural and functional properties of QA. This information has important implications for optimizing quinoa protein production, and various products related to quinoa protein could be developed. which provides the gist of commercial applications of quinoa seeds for spreading out in the marketplace.

## Introduction

Quinoa (*Chenopodium quinoa Willd.*) is a pseudo-cereal originating from the Andes (1). In recent years, quinoa has received tremendous attention, not only in South America but also worldwide, owing to its extensive adaptability to different environmental conditions and its versatile uses and nutritional value (2). Quinoa contains abundant levels of lipids, fiber, B vitamins, and minerals, which have functions of enhancing immunity, hypolipidemic, hypoglycemic, antioxidant, and anti-inflammatory effects (3). This high nutritional value and the gluten-free nature of quinoa make it useful for the development of a wide range of nutritious food items for individuals with celiac disease (4). Hence, quinoa is also called “the food of the future” and “plant gold” and is considered one of the most important crops for addressing food safety in the 21st century (5).

The nutritional value of foods primarily depends on their protein content and quality (6). The average protein content of quinoa ranges from 12% to 23% (7), higher than that of rice (8.8%), corn (10.5%), barley (11.0%), rye (11.6%), sorghum (12.4%), and wheat (14.8%), and close to that of beef (20%) (8). For grain cereals, another important indicator of nutritional value is the amino acid (AA) composition (9). Unlike most cereals, which are particularly lacking in Lys content (10), quinoa has an excellent AA balance (6). As such, it is among the few plant foods that provides all the essential amino acids needed by humans.

Quinoa protein is primarily made up of type 11S globulin and type 2S albumin, which account for approximately 37% and 35% of the protein content, respectively (7). Among them, the AA profile of the albumin fraction is more balanced and meets the FAO/WHO requirements (11), being rich in Cys, His, Arg, and sulfur-containing AAs. Therefore, it has great potential as a dietary addition, particularly for children, in the form of quinoa grains, quinoa derivatives, or quinoa-based dietary supplements (12). Further, albumin is well-known for its excellent emulsifying and foaming properties (13). Therefore, owing to its superior nutritional and functional properties, albumin has a broad range of prospective practical applications (14). However, since quinoa albumin (QA) is a still a relatively new source of food protein, research on QA has so far been limited, restricting its wide application in the food industry.

Heating is a prominent unit operation involved in quinoa processing and helps meet the safety and sensory needs of consumers. However, heating inevitably causes protein denaturation and structural changes and sometimes affects the functional and digestion properties of proteins, leading to substantially different physicochemical properties in the end product. Therefore, in order to guide the applications of proteins in the food industry, it is important to examine the effects of heat treatment on their structure, function, and digestion characteristics. Recent studies have shown that the structural and physical properties of proteins, including album protein isolates, lotus protein, oat protein, rice bran protein isolates, buckwheat globulin, and rice bran albumin, are dramatically affected by heat treatment (15–19). However, to our knowledge, the effects of heat treatment on the structure, function, and *in vitro* digestion characteristics of QA remain unclear.

Hence, in this study, QA was subjected to different degrees of heat treatment (50, 60, 70, 80, 90, 100, and 121°C) in order to determine the effect of heat on its functional and structural properties. Further, an *in vitro* digestion model was established to investigate the digestibility of heat-treated QA. This study focused on exploring the relationship between heat treatment and QA properties, which could be useful for its further application and processing in the food industry.

## Material and methods

### Materials

Quinoa (cv. Longli No. 1) was obtained from the Gansu Academy of Agricultural Sciences. The quinoa seeds were thoroughly cleaned manually 3 times with deionized water to remove impurities and saponins. Thereafter, the clean seeds were dried at 40°C until their moisture level reached 13 ± 1 g/100 g. Then, they were smashed and filtered through 60-mesh sieves. The obtained quinoa powder was sealed in valve bags until further use. The reagents used in this study were all of analytic grade.

### Sample preparation

The experimental protocol used for sample preparation was based on the study by Luo et al. (20), with minor modifications. Briefly, the quinoa powder was weighed and added to an equal volume of n-hexane (w/v) and stirred 4 times (48 h each) using a magnetic stirrer to remove flavonoids and lipids. Then, the solvent was evaporated under vacuum. The pre-treated powder (100 g) was collected and dissolved in 1,000 ml of deionized water, magnetically stirred at 4°C for 2 h, and then centrifuged at 13,500 × *g* for 25 min to eliminate any insoluble material. The pH was lowered to the isoelectric point of 3.4, which was identified via isoelectric focusing in pre-test experiments, and the albumin was precipitated. Subsequently, the precipitate was washed twice with deionized water, and the pH was adjusted to 7.0 using NaOH. The sample was centrifuged again, and the precipitate was collected. Following this, a concentrated extract was obtained as the crude extract of QA by freeze-drying after dialysis at 4°C for 72 h. The lyophilized powder of QA (2 g) was dissolved in 50 ml of 0.01 M phosphate buffer solution (PBS, pH 9.0). The sample was applied directly to a diethylaminoethylcellulose-52 (DEAE-52) column (1.0 × 10 cm) which had been previously equilibrated with 20 mM Tris−HCl buffer (pH 8.0). Unadsorbed protein fractions were collected with the equilibration buffer while adsorbed proteins were eluted by addition of 0.5 M NaCl in the buffer. The protein eluates were collected at a flow rate of 22.5 cm^3^ h^–1^ and the elution profile was monitored at 280 nm (A280), and then freeze-dried. All steps were performed at 4°C.

### Heat treatment

Before the analyses reaction, Lyophilized QA powders (10 g) were dispersed in 1000 mL PBS (0.2 M pH 7.0), preparing dispersions of concentrations ranging between 10 mg/ml (w/v) and then stirred magnetically at 25°C for 8 h for complete hydration. The solutions were heated in a water bath at 50, 60, 70, 80, 90, 100, and 121°C for 30 min (the temperature changes were detected using a temperature logger). After the heat treatments, all the samples were directly cooled down in an ice bath, the samples were stored at 4°C until analysis. Typically, all analyses were performed within 48 h.

### SDS−PAGE

Quinoa albumin was analyzed using SDS−PAGE (12% acrylamide separating gel and 5% acrylamide stacking gel containing 10% SDS) as described by Xiong et al. (21). Briefly, a QA dispersion (0.2 mM) was obtained directly in sample buffer [10 mM Tris−HCl, 10% (w/v) glycerol, 0.02% (w/v) bromophenol blue, 2% (w/v) SDS, and 5% (v/v) β-mercaptoethanol, pH 8.0]. The samples were boiled at 90°C for 10 min and then centrifuged at 1,500 × *g* and 4°C for 10 min. A BioRad protein standard (molecular weight, 10 to 200 kDa) was used as the marker. The gel was stained with Coomassie Brilliant Blue and decolorized with 10% acetic acid. Finally, the bands were photographed using an imaging system.

### Amino acid

The AA assay was performed as described by Machado et al. (22). First, 0.1 g of lyophilized QA powder was dissolved in 10 ml HCl (6 M) in a digestion tube, which was subsequently purged with nitrogen for 5 min. Before the analysis, digestion was conducted in vacuum-sealed hydrolysis tubes at 110°C (22 h). Tryptophan contents were measured following alkaline hydrolysis under the same conditions. After cooling, the digested sample was topped to 25 ml, and the digest (2 ml) was obtained and dried at 60°C for 24 h. The residue was dissolved in 2 ml of ultrapure water and dried again; this operation was repeated 3 times. The samples were collected and diluted to 5 ml. Then, 2 ml of the dilution was filtered through a 0.22-μm aqueous membrane and subsequently tested using an automated AA analyzer (LA8080; Hitachi Co., Tokyo, Japan), and the separation of the amino acid (AA) was carried out on a protein hydrolysate analysis column at a flow rate of 0.40 ml/min. The amino acids were identified and quantified by comparing the peak profiles of the samples with the standard AA profiles.

### Structure of quinoa albumin

#### Sulfhydryl groups and disulfide bonds

The sulfhydryl group and disulfide bond content were determined using Ellman’s reagent according to the method described by Pan et al. (23). Elman’s reagent was obtained by dissolving 40 mg of 5,5′-dithiobenzoic acid (DTNB) with 10 ml Tris–Gly buffer solution (1.2 g of disodium ethylene diamine tetraacetic acid [EDTA], 6.9 g of Gly, and 10.4 g of Tris in 1,000 ml of distilled water; pH 8.0). Lyophilized samples obtained after different treatments were dissolved in Tris-Gly-SDS buffer (90 ml Tris-Gly buffer containing 10 ml of SDS aqueous solution), bringing the final protein concentration to 10 mg/ml. The samples were lysed and centrifuged at 8,000 × *g* for 20 min, following which 4 ml of the supernatant was mixed with 0.04 ml of Ellman’s reagent and incubated in the dark for 30 min. Finally, the absorbance was measured at 412 nm using a spectrophotometer to determine the free sulfhydryl content. To examine the total sulfhydryl content, lyophilized protein samples were dissolved in a urea-Tris-glycine buffer containing (10 M urea, 2.5% SDS, 0.004 M EDTA, 0.09 M glycine, and 0.086 M Tris; pH 8.0), mixed evenly, and incubated for 30 min. Subsequently, the samples were centrifuged at 8,000 × *g* for 20 min, and the supernatant was dissolved in 5 ml of 12% TCA and centrifuged again. This wash step was repeated 3 times. The precipitate was collected and dissolved in 2.5 ml of Tris-glycine buffer, followed by the addition of 50 ml DTNB and incubation for 30 min under dark conditions. The overall content of sulfhydryl groups was detected based on the absorbance at 412 nm. The molar extinction factor was 1360 M^–1^cm^–1^. Additionally, the content of the disulfide bonds was obtained by subtracting the free sulfhydryl level from the total sulfhydryl level and dividing it by 2. The levels of sulfhydryl groups and disulfide bonds were calculated as follows:


(1) $$S\,H\,\left(\mu \,m\,o\,l\,/\,g\right)=\frac{75.53\times D\times A_{412}}{C}$$

(2) $$S\,S\,\left(\mu \,m\,o\,l\,/\,g\right)=\frac{C_{t}-C_{f}}{2}$$

where D is the coefficient of dilution; A_412_ indicates the absorbance of QA in Ellman’s reagent; C_*t*_ is the content of total sulfhydryl groups; and C_*f*_ is the content of the free sulfhydryl groups.

### Surface hydrophobicity

The Hydrophobicity (*H*_0_) was measured using the bromophenol blue (BPB) binding assay, as described by Zhang et al. (24). First, 200 μl of BPB (1 mg/ml) was added to 1 ml of a QA suspension (concentration adjusted to 5 mg/ml using PBS at pH 7) and mixed thoroughly. Blanks consisted of BPB (200 μl) and PBS (1 ml). These were stirred for 10 min and centrifuged at 2,000 × *g* for 15 min. The absorbance of the supernatant was then measured at 595 nm.


(3) $$B\,P\,B\,\,b\,o\,u\,n\,d\,\left(\mu \,g\right)=200\,\mu \,g\times \left(A_{\text{control}}-A_{\text{sample}}\right)\,/\,A_{\text{control}}$$

### Functional properties of quinoa albumin

#### Protein solubility

The solubilities of the samples were analyzed as described by Abugoch and Romero (25). In brief, the prepared 1% w/v QA suspensions were magnetically stirred for 1 h and centrifuged at 7,500 × *g* for 20 min. Bradford’s method was used for measuring the amount of QA in the supernatant. Solubility was indicated as a percentage of the total albumin.

#### Water and oil binding capacity

The water (WBC) and oil binding capacity (OBC) of QA were measured based on the methods used by Beuchat (26). The WBC was calculated as the grams of water retained per gram of sample, and the OBC was measured as the amount of oil held per gram of sample.

#### Emulsifying activity and emulsion stability

Emulsifying activity (EA) and emulsion stability (ES) assays were conducted according to the methods used by Zhang et al. (27). First, 15 ml of QA solution (10 mg/ml) was mixed with 5 ml corn oil using a homogenizer at 23,000 rpm for 2 min. Then, 2 ml of the homogenized sample was mixed with deionized water (18 ml), from which 0.5 ml was removed and mixed with 4.5 ml of 0.1% SDS. The absorption was measured at 500 nm using 0.1% SDS as the control:


(4) $$\text{EA}\,\left(\frac{m^{2}}{g}\right)=\frac{2\times 2.303\times A_{0}\times D\,F}{C\times \varphi \times \left(1-\theta \right)\times 10000}$$

(5) $$\text{ES}\,\left(\min\right)=\frac{A_{0}}{A_{0}-A_{30}}\times 100$$

where A_0_ is the sample absorbance value; DF represents the fold dilution (100); C is the emulsion solution that had a concentration before use (g/ml); φ is the oil volume fraction; θ is the oil phase fraction of 0.25; and A_30_ represents the absorbance at 30 min.

#### Foaming capacity and foam stability

Foaming capacity (FC) and foam stability (FS) measurements were adapted based on the protocol described by Aydemir and Yemenicioglu (28). First, 20 ml of 1% QA solution obtained after different treatments were homogenized at 23,000 rpm for 2 min. The mixture was poured into a 50-ml measuring cylinder, and the volume was recorded. The FC and FS were calculated according to the following formulae:


(6) $$FC\left(\%\right)=\left(\frac{V_{0}-5}{5}\right)\times 100$$

(7) $$FS\left(\%\right)=\left(\frac{V_{10}-5}{V_{0}-5}\right)\times 100$$

where V_0_ is the volume prior to homogenization (ml) and V_10_ is the volume after homogenization (ml).

### *In vitro* simulation of the gastrointestinal digestion of quinoa albumin

#### Preparation of simulated gastric fluid and simulated intestinal fluid

Simulated gastric fluid (SGF) and simulated intestinal fluid (SIF) were prepared according to the protocol detailed by Minekus et al. (29). In brief, SGF was prepared by mixing 0.7 ml HCl (12 M) with 2.0 g NaCl and 0.3 g pepsin to a final volume of 100 ml (pH 1.2). SIF was prepared by completely dissolving 0.68 g KH_2_PO_4_ in 25 ml deionized water, and later adding 19 ml NaOH (0.2 M) and 40 ml deionized water, and finally 4.0 g trypsin (pH 7.5).

#### *In vitro* gastrointestinal digestion

*In vitro* digestion was performed as described by Cho (30). QA solution (5 mg/ml) and an equivalent amount of SGF were placed in a centrifuge tube and shaken in a 37°C water bath for 30, 60, 90, and 120 min. The digests were immediately boiled at 100°C (10 min) to kill the enzymes and then cooled quickly on the ice. After adjusting the pH of the remaining solution to 7.5, an equivalent amount of SIF was added and shaken at 37°C. The digestion products were removed at 150, 180, 210, 240, 270, and 300 min, boiled at 100°C, and then cooled and set aside.

#### Degree of hydrolysis

O-phthalaldehyde (OPA) was used to determine the degree of QA hydrolysis (31). For this test, 400 μl of hydrolyzed supernatant was mixed with 3 ml OPA, and after 2 min of incubation, the absorbance at 340 nm was measured. A serine standard solution (0.9516 mM) and deionized water were employed as the standard and blank, respectively. To calculate the degree of hydrolysis, the following formulae was used:


(8) $${S\,e\,r\,i\,n\,e\,N\,H}_{2}=\frac{A_{s\,a\,m\,p\,l\,e-}A_{b\,l\,a\,n\,k}}{A_{s\,t\,a\,n\,d}-A_{b\,l\,a\,n\,k}}\times 0.9516\times \frac{N\times V}{X\times P}$$

where SerineNH_2_ is the amount of Ser per each g protein (mmol/g); X is the sample quality (g); p is the sample protein content (%); N is the number of dilutions; and V is the supernatant volume (L).


(9) $$DH\left(\%\right)=\frac{\left({S\,e\,r\,i\,n\,e\,N\,H}_{2}-\beta \right)\,/\,\alpha }{h_{t\,o\,t}}\times 100$$

where α and β represent the constants 1 and 0.4, respectively, and the h_*tot*_ of QA is 7.4 mmol/g.

#### Total amino acid content of the *in vitro* digestion products

The digestion products were lyophilized, and the method for AA analysis was identical to the AA analysis method described previously (section “Amino acid”).

### Scanning electron microscopy

Quinoa albumin was observed and analyzed morphologically using SEM. Freeze-dried samples were sputtered with gold and observed on a Hitachi S-3400N scanning electron microscope (Hitachi, Tokyo, Japan) at an acceleration potential of 20.0 kV.

### Statistical analysis

The results are expressed as the mean ± standard deviations (SD). Data were analyzed with Duncan through multiple comparisons and one-way analysis of variance (ANOVA) using SPSS Win 27.0 software (SPSS Inc., Chicago, IL, United States). Statistical significance was set at *P* < 0.05.

## Results and discussion

### SDS−PAGE

**FIGURE 1:**
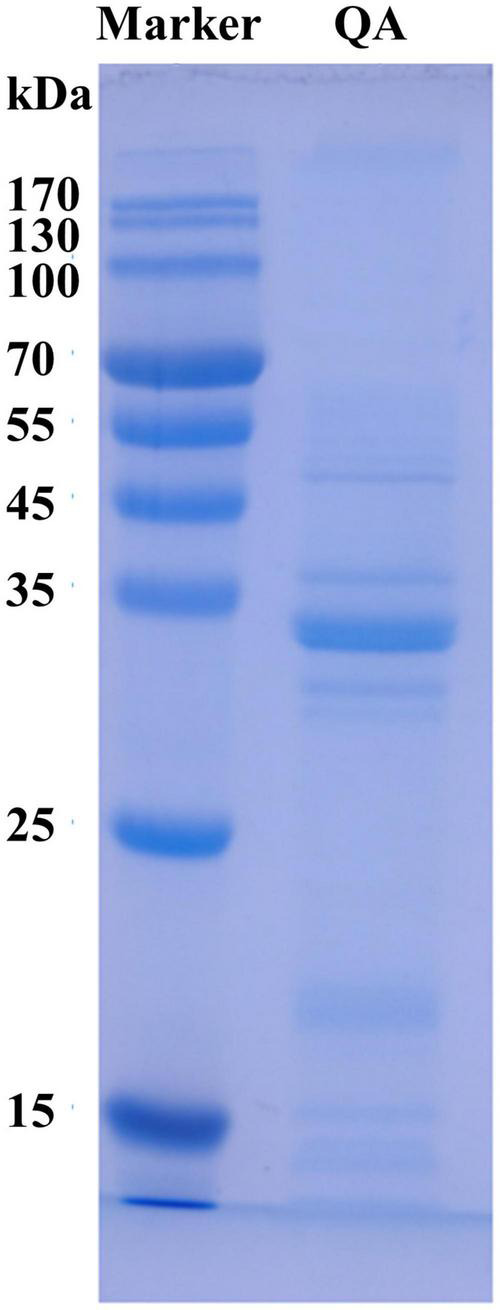
SDS–PAGE profile of quinoa albumin. QA, quinoa albumin.

The molecular weight of a protein is highly associated with its functional properties, and especially its ability to form gels or emulsions (32). The electrophoresis findings are displayed in Figure 1. Following SDS−PAGE, QA showed strong bands with molecular weights of 20–35 kDa and few bands with molecular weights >40 kDa. Some faint bands of <25 kDa were also observed. This was consistent with a previous study on kidney bean albumin, which was found to contain protein subunits sized 30 kDa and main polypeptide bands of 27 kDa (33).

### Amino acid composition

Proteins are mainly composed of AAs that act as auxiliary ligands (34). The AAs found in QA were compared to the FAO/WHO models of reference, as shown in Table 1. In QA, the content of branched-chain amino acids (BCAAs) — which are valuable for anti-hypertensive drug development (35) — was approximately 2-fold that of the standards stipulated by FAO/WHO (*P* < 0.05). Negatively charged amino acids (NCAAs) such as Asp and Glu have extra electrons that enhance the reducing properties of iron, preventing iron-induced lipid oxidation and food oxidative damage (35). As a result, a higher NCAA level increases the antioxidant effect of albumin. Compared with the FAO/WHO standard, QA had a significantly greater Arg/Lys ratio (1.27), giving it a competitive advantage associated with reduced cholesterol and improved cardiovascular health (36). Aromatic amino acids (AAAs), the building blocks of proteins, have an important role in secondary metabolism. The amount of AAAs in QA exhibited about 2.37-fold higher than that described by FAO/WHO standards. Additionally, QA showed an approximately 2-fold enhancement of positively charged amino acids (PCAAs) — which are well-recognized determinants of membrane protein topology — when compared with the FAO/WHO standard.

**TABLE 1:** Amino acid composition of quinoa albumin in comparison to the WHO/FAO standard.

Items (g/100 g protein)	Quinoa albumin	FAO/WHO pattern
**Essential amino acids**		
His	1.50 ± 0.07	1.60
Leu	4.67 ± 0.21	1.90
Ile	2.66 ± 0.12	1.30
Lys	3.72 ± 0.17	1.60
Met	0.69 ± 0.02	1.70
Cys	0.28 ± 0.01	
Phe	3.01 ± 0.14	1.90
Tyr	1.99 ± 0.09	
Thr	2.29 ± 0.11	0.90
Val	2.71 ± 0.11	1.80
Try	0.68 ± 0.04	0.50
**Non-essential amino acids**		
Ala	2.71 ± 0.13	0.26
Gly	2.62 ± 0.12	0.20
Pro	2.85 ± 0.02	0.61
Ser	3.09 ± 0.16	0.53
Glu	13.13 ± 0.58	1.75
Asp	7.27 ± 0.33	0.88
Arg	4.74 ± 0.23	0.46
Total essential amino acids	24.21 ± 1.09	13.20
Total non-essential amino acids	36.41 ± 1.57	17.89
Total amino acids	60.62 ± 2.66	4.69
Aromatic amino acids	5.69 ± 0.27	2.40
Branched-chain amino acids	10.04 ± 0.44	5.00
Negatively charged amino acid residues	5.22 ± 0.24	3.20
Positively charged amino acids	9.96 ± 0.47	3.66
Arg/Lys	1.27 ± 0.00	0.29

### Structure of quinoa albumin

#### Sulfhydryl group and sulfhydryl bond contents

**FIGURE 2:**
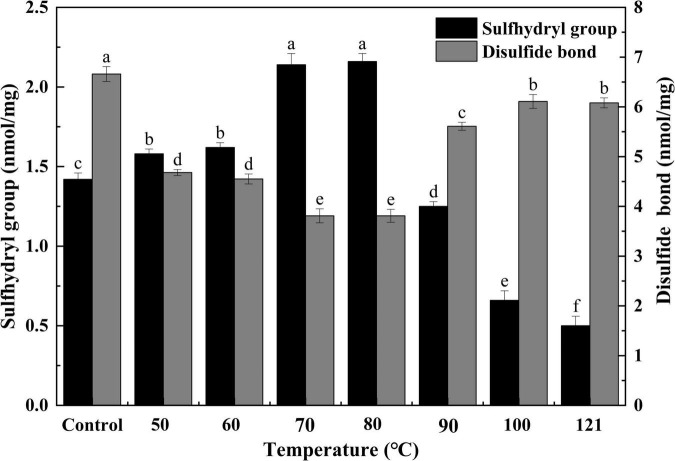
Effect of heat treatment on sulfhydryl and disulfide bonds in quinoa albumin. Superscripts (a–f) represent significant differences at the *P* < 0.05 level.

Sulfhydryl groups function as precursors of disulfide bonds, which are extremely important for the tertiary structure of a protein. Typically, an increase in sulfhydryl content indicates alterations to a protein’s structure (37). Therefore, sulfhydryl and disulfide bond analysis were indispensable for evaluating the structural and functional changes in QA (38). As shown in Figure 2, with increasing temperature, the content of sulfhydryl groups first increased and then decreased (*P* < 0.05). Moreover, the opposite trend was observed for disulfide bonds (*P* < 0.05). The highest content of sulfhydryl groups (2.16 nmol/mg) and lowest content of disulfide bonds (3.18 nmol/mg) were found at 80°C. The above data indicated that moderate heating (50–80°C) could increase the sulfhydryl group content while reducing the disulfide bonds in QA. This could be due to the unfolding of protein structure under heat treatment and thus the subsequent exposure of interior sulfhydryl groups. When protein subunits dissociate, disulfide bonds are broken and sulfhydryl is generated (39). Nevertheless, the opposite trend was the case under too high-temperature treatment that QA may undergo partial dissociation of protein aggregates, exposing more disulfide bonds. The changes in SH of heated-QA were similar to the irradiated quinoa albumin. The SH content of rice albumin increased significantly as the irradiation dose, this tendency could be ascribed to its higher sulfur-containing amino acids (i.e., methionine) and more disulfide bonds destructed by irradiated (40). Combining the result of increased SH content, it was supposed that disulfide bonds of proteins were destroyed under heat treatment and then exposed to the solution, which contributed to the solubilization.

#### Surface hydrophobicity

**FIGURE 3:**
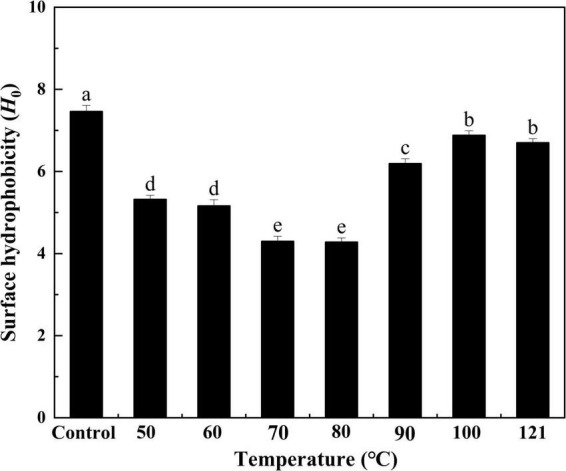
Effect of heat treatment on the surface hydrophobicity of quinoa albumin. Superscripts (a–e) represent significant differences at the *P* < 0.05 level.

Hydrophobicity indicates the degree of exposure to surface hydrophobic groups and is closely associated with the interfacial activity of a protein (41). As seen in Figure 3, heat treatment had a significant effect on the *H*_0_ of QA. Specifically, *H*_0_ values initially decreased with increasing temperatures and then increased thereafter (*P* < 0.05), reaching the lowest value of 4.28 at 80°C (approximately 57% of the control). Notably, the comparable trend was detected for *H*_0_ changes of flaxseed albumin at different pH conditions, this phenomenon could be attributed to the variations in molecular interaction in the protein molecules, leading to denaturation of protein molecules (42). This lower hydrophobicity could be due to the fewer charges on the protein surface, which causes protein aggregation, thus burying hydrophobic sites in the aggregated structures following heat treatment (43). In contrast, when the temperature is too high, protein molecules can unfold, exposing buried hydrophobic groups and resulting in increased hydrophobicity (44). Likewise, similar results were obtained when the hydrophobicity of protein isolates from heat-treated legumes was compared with that from the untreated group (45).

### Functional properties

#### Solubility

**FIGURE 4:**
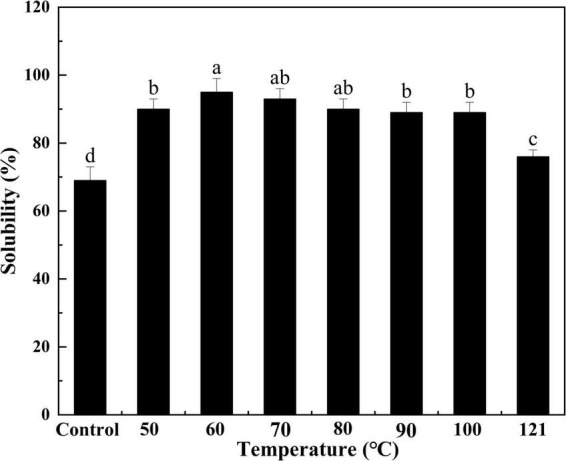
Effect of heat treatment on the solubility of quinoa albumin. Superscripts (a–d) represent significant differences at the *P* < 0.05 level.

Solubility is a measure of the degree of protein aggregation and denaturation, as well as an indicator of protein functionality (46). As depicted in Figure 4, the solubility of QA gradually increased with increasing temperature (*P* < 0.05), achieving a peak of 95% at 60°C. It then declined progressively as the temperature continued to rise (*P* < 0.05). Protein solubility is determined by the interactions between protein and water molecules, on the one hand, and the aggregated properties of protein fraction in the process of heating, on the other hand (47). Hence, the appropriate conditions of heating can promote the hydration capacity of a protein and further increase its solubility. Yet, once the temperature becomes too high, the protein denatures, the conformation of the protein molecule changes, and the hydrophobic groups present in the protein are exposed, leading to reduced protein solubility. This trend is similar to that observed from the solubility of the chicken bone protein in ultrasonic power study, possibly due to the cavitation and thermal effects that increase the energy of molecules in the system (48).

#### Water binding capacity and oil binding capacity

**FIGURE 5:**
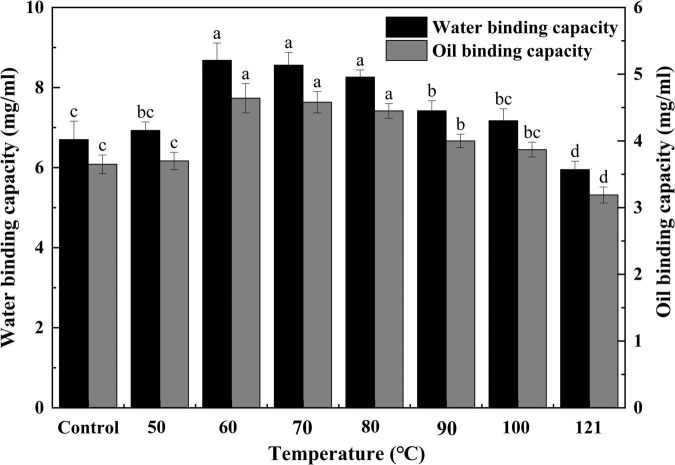
Effect of heat treatment on the water and oil binding capacity of quinoa albumin. Superscripts (a–d) represent significant differences at the *P* < 0.05 level.

The WBC and OBC of a protein are crucial metrics to be considered while formulating food products (49). The mouthfeel and texture of foods are affected by the WBC, and the OBC is associated with the emulsion properties of products such as mayonnaise (50). As shown in Figure 5, the strength of water binding in QA increased progressively with a rise in temperature, but after 60°C, a gradually decreasing trend was observed (*P* < 0.05). Variations in WBC may be caused by the thermally induced unwinding of protein segments that occurs when the temperature increases, and there is an increase in the hydrophilic groups on the surface, enhancing the interaction force. On the contrary, when the temperature rises beyond a certain threshold, the aggregation of denatured proteins may lead to a decrease in their ability to bind to water. The OBC of QA tended to first rise and then fall with increasing temperature (*P* < 0.05), with the maximum value being reached at 60°C. This was probably due to the near-complete unfolding of the proteins during the process of heating, which increases the interaction between the small molecules and the oil, thereby expanding the OBC. Nevertheless, the oil’s viscosity decreases, and fluidity increases with a further increase in temperature, resulting in a weaker OBC. Similar types of results for WBC and OBC were observed in heat-treated sunflower protein isolates (44). The improvement in WBC and OBC were also noted in nano fibrillated whey protein isolates after the application of heat treatment (51). Thus, changes in protein conformation with increasing binding sites under appropriate heating could lead to better WBC and OBC functionality (52). Heat treatment provides fresh ideas for included baked goods, cereals, ice creams, various dessert formulations, and chocolates.

#### Emulsifying activity and emulsion stability

**FIGURE 6:**
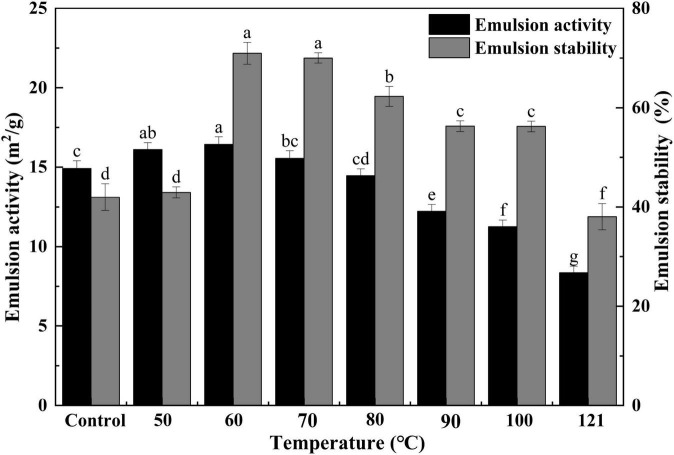
Effect of heat treatment on the emulsifying activity and emulsion stability of quinoa albumin. Superscripts (a–f) represent significant differences at the *P* < 0.05 level.

Generally, EA functions to measure the capacity of interfacial adsorption onto the oil-water interface of proteins, and ES is employed to examine the protein’s ability to be retained on an oil-water interface after storage in an emulsion (53). To evaluate the emulsion properties of QA, its EA and ES were measured in this study. As shown in Figure 6, a similar trend was observed in EA and ES variation. Both values first increased with temperature, peaking at 60°C, and then tended to decrease thereafter (*P* < 0.05). There is evidence of a tight relationship between the solubility and emulsification properties of a protein (54). In the present study, the emulsification ability of QA was markedly affected by heat treatment, probably as a result of the increased solubility, which allowed the rapid spreading and adsorption of QA at the oil-water interface (46) and thus elevating its emulsification capacity (55). A similar kind of observation was obtained for the emulsion activity and stabilities of heat-treated vanillin-rich protein isolates from kidney beans (45). The emulsifying activities of proteins are affected by their molar mass, hydrophobicity, conformational stability, charge and physicochemical factors such as pH, ionic strength, and temperature (56), in which, solubility is regarded as a prime factor for the emulsifying properties of protein isolates. Steaming and baking exert negative impacts on the emulsion stability of quinoa protein isolate, which might be related to the aggregation of protein molecules and the reduction of viscosity (57).

#### Foaming capacity and foam stability

**FIGURE 7:**
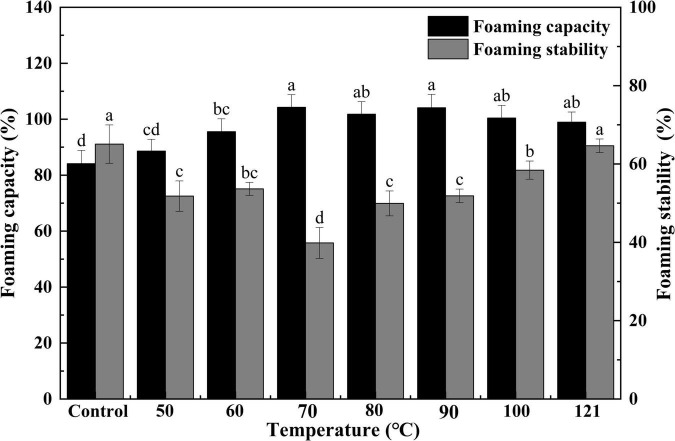
Effect of heat treatment on the foaming capacity and foam stability of quinoa albumin. Superscripts (a–d) represent significant differences at the *P* < 0.05 level.

Foaming capacity represents the size of the interfacial amount produced by a protein during the process of foaming, while FS represents the ability of a protein to stabilize bubbles under the influence of gravity (44). The FC and FS of QA after different heat treatments are presented in Figure 7. A gradual and continuous elevation of FC was observed with an increase in treatment temperatures (*P* < 0.05), but this value began to plateau after 70°C (*P* > 0.05). During the process of heating, protein chains are partially unraveled. This allows them to be more easily absorbed at the air-water interface, thus increasing FC (58). Additionally, FS exhibited an initial decrease, reaching its minimum at 70°C, and then tended to increase with increasing temperatures subsequently. The improvements in FS may be the result of increased protein-protein interaction (aggregation) during heating, leading to the formation of a thick protein membrane surrounding the produced bubbles (59). Ding et al. (60) found that the trend of FC under other physical treatments was similar to that of heat treatment, which showed an upward trend with increasing intensity of physical conditions, followed by a downward trend. At the same time, we found that the heated albumins with over 85% of FC which was significantly higher than the corn germ meal albumin of FC (67%) (61).

### *In vitro* digestion of quinoa albumin

#### Degree of hydrolysis during *in vitro* digestion

**FIGURE 8:**
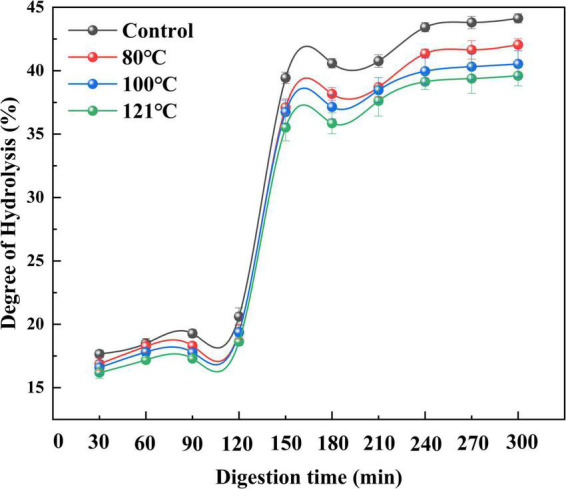
Effect of heat treatment on the degree of hydrolysis of quinoa albumin during *in vitro* digestion. Error bars represent standard deviations. Results are the means of triplicate values.

An *in vitro* digestion assay was conducted by simulating artificial gastrointestinal fluid. Changes in the hydrolysis rate represent the degree of peptide bond cleavage during simulated digestion (62). As illustrated in Figure 8, the degree of hydrolysis of QA increased with prolongation of digestion time (*P* < 0.05). Moreover, heat-treated groups (80, 100, and 121°C) showed lower degrees of hydrolysis than the control, with a similar tendency across the process of digestion. This is compatible with findings from Tian et al. (63) which showed that the degree of hydrolysis of soy protein isolates reduces after thermal treatment above 85°C. Similarly, Sangsawad et al. (64) also reported that high-temperature treatment at 121°C strongly decreases the digestibility of chicken breast protein. The change in QA digestibility after heat treatment can be attributed to cross-linked protein aggregates, which exhibit good resistance to hydrolysis (65). However, these results were found to be the opposite of those of germinated legume proteins after soaking treatment (66). High-pressure processing treated pea protein underwent a greater degree of proteolysis and showed different peptide patterns after static gastric digestion compared to untreated and heat-treated pea protein (67).

#### Total amino acid content during *in vitro* digestion

**FIGURE 9:**
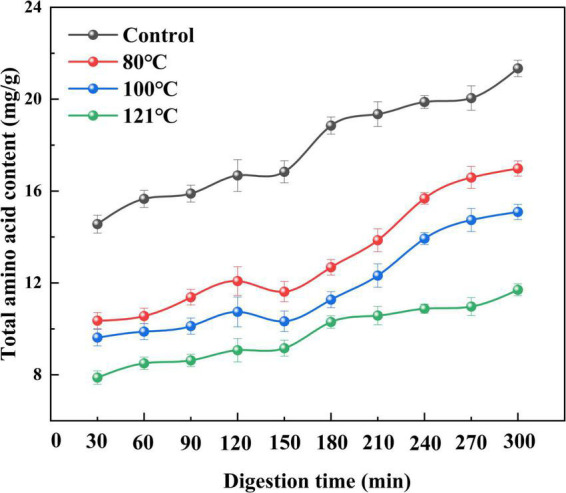
Effect of heat treatment on the total amino acid content of quinoa albumin during *in vitro* digestion. Error bars represent standard deviations. Results are the means of triplicate values.

Changes in the total AA content of QA during digestion following different treatments are shown in Figure 9, and the classification of the AAs can be seen in Table 2. The total AA levels at the end of trypsin digestion (300 min) were remarkably higher than those at the end of pepsin digestion (120 min) irrespective of treatment temperature (*P* < 0.05). Moreover, the content of AAs in the *in vitro* digestion product of unheated QA was higher than that in heat-treated samples (*P* < 0.05). Among them, the AA content after heat treatment at 121°C was the lowest (11.70 mg/g) at the end of digestion, 45.1% lower than the control. These results are consistent with those from Shi et al. (68) which demonstrated that heat treatment significantly reduces the AA content of apricot mushroom proteins following *in vitro* digestion. Similar findings have also been reported regarding lower AA levels following the *in vitro* digestion of bran proteins after microwave heat treatment (69). The extent of protein digestion in the gastrointestinal tract can be indicated by the levels of Arg, Lys, Phe, Trp, and Tyr, which are the targeted cutting sites for pepsin and pancreatic proteases (70). Our experimental results support the findings that heating reduces the content of Arg, Lys, Phe, Trp, and Tyr in QA (*P* < 0.05). As a result, we speculate that high-temperature thermal treatment may cause severe destruction to AAs and induce irreversible decomposition, leading to a loss of contents and finally a reduced hydrolysis ratio. Joycelyn et al. (71) found that heat-induced protein unfolding led to a 38% decrease in the amount of Cys in amino acid compared to untreated cowpea proteins, whereas combined thermal-ultrasonic treatment increased Cys content of the hydrolysates by 41%. This will be an interesting subject for future QA processing that heating technology could combine with non-heat ones to enhance digestibility.

**TABLE 2:** Total amino acid content of the *in vitro* digestion products of quinoa albumin after different heat treatments.

Items	Content (g/100 g)							
	
	Control		80°C		100°C		121°C	
				
	120 min	300 min	120 min	300 min	120 min	300 min	120 min	300 min
**Essential amino acids**								
His	0.72 ± 0.03^A^	0.92 ± 0.03^a^	0.52 ± 0.02^B^	0.73 ± 0.03^b^	0.46 ± 0.02^BC^	0.65 ± 0.01^c^	0.39 ± 0.02^C^	0.54 ± 0.02^d^
Leu	1.69 ± 0.11^A^	2.16 ± 0.08^a^	1.28 ± 0.06^B^	1.72 ± 0.08^b^	1.09 ± 0.07^C^	1.53 ± 0.07^bc^	0.92 ± 0.05^D^	1.18 ± 0.04^c^
Ile	0.14 ± 0.01^B^	0.18 ± 0.01^ab^	0.16 ± 0.01^AB^	0.14 ± 0.01^b^	0.19 ± 0.02^A^	0.22 ± 0.01^a^	0.17 ± 0.01^AB^	0.21 ± 0.02^ab^
Lys	0.16 ± 0.01^A^	0.22 ± 0.02^a^	0.15 ± 0.01^A^	0.17 ± 0.01^b^	0.14 ± 0.01^AB^	0.16 ± 0.01^b^	0.13 ± 0.01^B^	0.14 ± 0.01^b^
Met	0.42 ± 0.02^A^	0.54 ± 0.03^a^	0.3 ± 0.02^B^	0.43 ± 0.02^b^	0.27 ± 0.02^B^	0.38 ± 0.02^bc^	0.23 ± 0.01^C^	0.29 ± 0.02^c^
Cys	0.2 ± 0.01^A^	0.26 ± 0.01^a^	0.15 ± 0.01^B^	0.21 ± 0.01^b^	0.13 ± 0.01^BC^	0.18 ± 0.01^bc^	0.11 ± 0.01^C^	0.14 ± 0.01^c^
Phe	0.23 ± 0.02^A^	0.3 ± 0.02^a^	0.17 ± 0.01^B^	0.24 ± 0.01^b^	0.15 ± 0.02^BC^	0.21 ± 0.02^bc^	0.13 ± 0.01^C^	0.16 ± 0.01^c^
Tyr	0.67 ± 0.04^A^	0.85 ± 0.03^a^	0.48 ± 0.02^B^	0.68 ± 0.02^b^	0.43 ± 0.03^BC^	0.6 ± 0.01^bc^	0.36 ± 0.02^C^	0.47 ± 0.02^c^
Thr	2.94 ± 0.12^A^	3.34 ± 0.24^a^	2.56 ± 0.25^AB^	3.21 ± 0.06^ab^	2.04 ± 0.11^B^	2.57 ± 0.08^b^	1.14 ± 0.05^C^	1.97 ± 0.14^c^
Val	0.47 ± 0.02^A^	0.6 ± 0.04^a^	0.34 ± 0.02^B^	0.47 ± 0.02^b^	0.3 ± 0.01^BC^	0.42 ± 0.02^b^	0.25 ± 0.02^C^	0.33 ± 0.02^c^
Try	0.76 ± 0.04^A^	0.97 ± 0.03^a^	0.55 ± 0.02^B^	0.77 ± 0.04^b^	0.49 ± 0.02^BC^	0.69 ± 0.03^bc^	0.41 ± 0.02^C^	0.53 ± 0.02^c^
**Non-essential amino acids**								
Ala	0.84 ± 0.03^A^	1.07 ± 0.07^a^	0.61 ± 0.03^B^	0.85 ± 0.05^ab^	0.54 ± 0.02^C^	0.76 ± 0.04^b^	0.46 ± 0.03^D^	0.59 ± 0.02^c^
Gly	0.69 ± 0.03^A^	0.89 ± 0.05^a^	0.5 ± 0.02^B^	0.71 ± 0.03^b^	0.45 ± 0.02^BC^	0.63 ± 0.03^c^	0.38 ± 0.02^C^	0.49 ± 0.02^d^
Pro	0.58 ± 0.02^A^	0.74 ± 0.01^a^	0.42 ± 0.02^B^	0.59 ± 0.03^b^	0.37 ± 0.01^C^	0.52 ± 0.02^c^	0.31 ± 0.01^D^	0.41 ± 0.01^d^
Ser	1.22 ± 0.06^A^	1.56 ± 0.09^a^	0.88 ± 0.06^B^	1.24 ± 0.06^b^	0.78 ± 0.04^BC^	1.1 ± 0.07^bc^	0.66 ± 0.03^C^	0.85 ± 0.03^d^
Glu	0.29 ± 0.01^A^	0.37 ± 0.02^a^	0.21 ± 0.01^B^	0.3 ± 0.02^b^	0.19 ± 0.01^B^	0.26 ± 0.02^bc^	0.16 ± 0.01^C^	0.21 ± 0.01^c^
Asp	1.84 ± 0.07^A^	2.35 ± 0.15^a^	1.33 ± 0.08^B^	1.87 ± 0.06^b^	1.18 ± 0.1^C^	1.66 ± 0.05^c^	1.02 ± 0.02^D^	1.29 ± 0.08^d^
Arg	1.93 ± 0.08^A^	2.47 ± 0.17^a^	1.4 ± 0.05^B^	1.96 ± 0.08^b^	1.24 ± 0.05^C^	1.74 ± 0.03^c^	1.05 ± 0.05^D^	1.35 ± 0.04^d^
**Total amino acids**	16.68 ± 0.23^A^	21.34 ± 0.31^a^	12.08 ± 0.21^B^	16.98 ± 0.31^b^	10.74 ± 0.15^C^	15.09 ± 0.22^bc^	9.07 ± 0.18^D^	11.71 ± 0.24^c^

Superscripts A—D indicate the amino acid levels showing a significant differences (P < 0.05) at the end of pepsin digestion.

Superscripts a—d indicate the amino acid levels showing a significant differences (P < 0.05) at the end of trypsin digestion.

### Scanning electron microscopy

**FIGURE 10:**
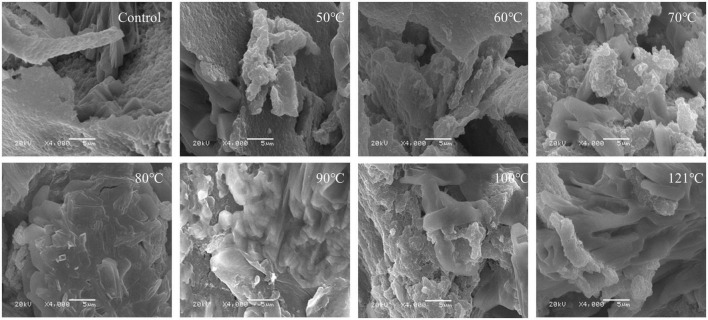
Morphological characteristics of heat-treated quinoa albumin.

Studies have revealed a close relationship between the microstructure and functional properties of proteins. SEM is a frequently used and reliable technique for characterizing protein microstructures (72). The SEM images of heated QA are depicted in Figure 10. The findings showed that after heat treatment, QA tended to become disordered and form irregular fragment structures. Additionally, a rougher surface with lamellar structure and lower porosity were visible in the untreated albumin lamina. Less variation in the albumin surface was observed between 50°C and 60°C, while QA dissociation and reaggregation resulted in the formation of tight aggregates, dense pore distribution, and larger pore sizes were detected with a continuous increase in temperature. Particularly, the variation was most evident at 121°C. This phenomenon likely occurs because of micelle dissociation when QA undergoes heat treatment. Moreover, higher temperatures lead to greater levels of dissociation. Similarly, it has been reported that the microstructure of corn gluten meal became compact and porous surface with small aggregates after ultrasonic treatment, possibly arising from protein-protein interaction or association of starch granules with protein molecules. Free–SH groups are deactivated during the heating or ultrasonication process, thus reducing aggregation of proteins, with the lamellar structure (73). These phenomena were also observed in individual egg white proteins after multiple freeze-thaw processing (74).

## Conclusion

In the current study, the effects of heat-treated at various temperatures on the structure, functional properties, and *in vitro* digestibility of QA were investigated. SDS−PAGE revealed that the subunits of QA had molecular weights ranging from 10 to 40 kDa. Heat treatment altered the molecular arrangements and interactions of QA, altering functional properties such as foaming, solubility, and emulsification via changes in sulfhydryl-disulfide bonds and surface hydrophobicity. Furthermore, heat treatment reduced the *in vitro* digestibility of QA in a temperature-dependent manner. Taken together, the findings suggest that heat treatment could be a valid method for improving the functional and nutritional properties of QA. It could also be used as a bioprocessing tool for quality improvements in quinoa seed proteins for food, nutritional health applications, and biopharmaceuticals.

## Data availability statement

The original contributions presented in this study are included in the article/supplementary material, further inquiries can be directed to the corresponding author/s.

## Author contributions

CY contributed to the conceptualization, investigation, data curation, software, and writing – original draft. XZ contributed to the methodology, and writing – review and editing. ZhaZ contributed to the data curation and reviewing. FaY and ZheZ contributed to the software and editing. YW contributed to the methodology, investigation, and editing. FuY contributed to the investigation, writing, and reviewing. All authors contributed to the article and approved the submitted version.
